# Frequency of loss of heterozygosity of the *NF2* gene in schwannomas from Croatian patients

**DOI:** 10.3325/cmj.2012.53.321

**Published:** 2012-08

**Authors:** Nives Pećina-Šlaus, Martina Zeljko, Hrvoje Ivan Pećina, Tamara Nikuševa Martić, Niko Bačić, Davor Tomas, Reno Hrašćan

**Affiliations:** 1 Laboratory of Neurooncology, Croatian Institute for Brain Research, School of Medicine University of Zagreb, Zagreb, Croatia; 2 Department of Biology, School of Medicine, University of Zagreb, Zagreb, Croatia; 3 Department of Radiology, Sestre Milosrdnice University Hospital, Zagreb, Croatia; 4 Ljudevit Jurak Department of Pathology, Sestre Milosrdnice University Hospital, Zagreb, Croatia; 5 Department of Biochemical Engineering, Faculty of Food Technology and Biotechnology, University of Zagreb, Zagreb, Croatia

## Abstract

**Aim:**

To identify gross deletions in the *NF2* gene in a panel of schwannomas from Croatian patients in order to establish their frequencies in Croatian population.

**Methods:**

Changes of the *NF2* gene were tested by polymerase chain reaction/loss of heterozygosity (LOH) using two microsatellite markers, D22S444 and D22S929.

**Results:**

The analysis with both markers demonstrated that 43.75% of schwannomas exhibited LOH of the *NF2* gene. The D22S444 region exhibited 45.5% of LOHs and the D22S929 region exhibited 14.3% of LOHs. Four LOHs were found in Antoni B, 2 in Antoni A, and 1 in Antoni A and B type tumors.

**Conclusion:**

The frequency of changes observed in Croatian patients is broadly similar to that reported in other populations and thus confirms the existing hypothesis regarding the tumorigenesis of schwannomas and contributes to schwannoma genetic profile helping us to better understand its etiology and treatment.

Schwannomas are benign encapsulated tumors of Schwann cells, the main peripheral glia cells that do not invade the nerve, but rather grow around it. It is extremely rare for a schwannoma to transform and become malignant (1,2). The majority of schwannomas arise spontaneously and only 4% are associated with neurofibromatosis type 2 (NF2). Sporadic schwannomas represent 6%-8% of all intracranial tumors. Additionally, schwannomas make up to 90% of tumors that occur in the cerebellopontine angle (3). During the last decade, great progress has been made in the determination of molecular and genetic characteristics causative of both sporadic and familial forms of schwannomas.

Schwannomas are a principal feature of two hereditary tumor diseases, NF2 and schwannomatosis. NF2 is an autosomal dominant disorder caused by germline mutations in the *NF2* gene on 22q12. The population-based birth incidence of NF2 was estimated as 1 case in 33 000-40 000 individuals (4,5). Approximately 50% of NF2 cases harbor mutations *de novo*, which cannot be identified in any other family members, and this suggests a high mutation rate for this gene (1,3). The hallmark of this disorder is the clinical finding of bilateral schwannomas involving the eighth cranial nerve (vestibular schwannomas) (6,7). Schwannomas also occur spontaneously, ie, sporadically. An annual incidence of sporadic vestibular schwannoma was approximately 1.3 per 100 000 (8). A population-based study in Denmark showed an estimated incidence of 11.5 cases per million inhabitants per year (9), while the US national tumor registry reported 1.1 cases per 100 000 people per year. Loss of heterozygosity, ie, gross deletion of the *NF2* gene is a common feature found in the majority of sporadic schwannomas. At present, it seems that all sporadic schwannomas are caused by some kind of alteration of *NF2* gene (10). The majority of detected deletions and mutations result in a truncated (shorter) protein products (11). The evidence very strongly suggests that all schwannomas are caused by changes in both gene copies and the consequent loss of NF2 protein function (12).

The main reason why we propose studying *NF2* gene in schwannomas is because there are still many unsolved and inadequately explained issues regarding the full genetic profile of human schwannomas. Today, it is recognized that alterations of the *NF2* gene are a causative event in the tumorigenesis of schwannomas. Therefore, identification of gross deletions of *NF2* gene in a set of patients from Croatia (the first time on a southeastern European population) can contribute to our knowledge of the total frequency of *NF2* alterations and thus improve our understanding of this tumor’s etiology.

## Materials and methods

### Tumor specimen

Samples of 20 schwannomas, together with autologous blood samples, were collected from the Department of Neurosurgery and Department of Pathology, Sestre Milosrdnice University Hospital, Zagreb, Croatia. The patients were without clinical NF1, NF2, or Schwannomatosis and had no family history of brain tumors. The schwannoma tissues were frozen in liquid nitrogen and transported to the laboratory, where they were immediately transferred at -75°C. The peripheral blood samples were collected in ethylenediaminetetraacetic acid (EDTA) and processed immediately.

Magnetic resonance imaging (MRI) revealed that the majority of schwannomas were intracranial, while two were located in the spinal nerves. During the operative procedure, the schwannomas were removed using a microneurosurgical technique. They were studied and classified according to WHO criteria by pathologists. Our study was approved by the ethics committees of Medical School University of Zagreb and Sestre Milosrdnice University Hospital, and the patients gave their informed consent.

### DNA extraction

Approximately 0.5 g of tumor tissue was homogenized with 1 mL extraction buffer (10 mM Tris HCl, pH 8.0; 0.1 M EDTA, pH 8.0; 0.5% sodium dodecyl sulfate) and incubated with proteinase K (100 μg/mL; Sigma, St. Louis, MO, USA; overnight at 37°C). Phenol chloroform extraction and ethanol precipitation followed.

Blood was used to extract leukocyte DNA. Five milliliters of blood was lysed with 7 mL distilled water and centrifuged (15-minute/5000 g). The pellet was processed as for DNA extraction from the tissue samples.

### Polymerase chain reaction

Two polymorphic regions, D22S444 and D22S929, of the *NF2* gene were studied. In a total volume of 25 μL, two polymorphic markers were amplified by using 5 pmol of each primer (Table 1), 200 ng DNA, 2.5 μL 10X buffer II (500 mM KCl, 100 mM Tris-HCl, pH 8.3), 1.5 mM MgCl_2_, 2.5 mM of each dNTP, 0.2 μL (1U) of *Taq* polymerase (Promega, Madison, WI, USA). PCR conditions: initial denaturation, 10 minutes/95°C; denaturation, 30 seconds/95°C; annealing, 30 seconds/55°C; extension, 30 seconds/72°C; final extension, 72°C/10 minutes; 35 cycles. All PCR products were analyzed on 2% agarose gels.

**Table 1:** Primers used for the amplification of microsatellite markers for the *NF2* gene

Marker	Primers (5′→ 3′)
D22S929	CTGCAGATCACAAACTCCTTG GCATTTATGGAGTATCCACAG
D22S444	TTTGAACTAAGCCTTAAAAATGC TGTTTGGCTTGAAGAAGGAG

### Loss of heterozygosity

To assess LOH of the *NF2 *gene, markers D22S444 (13,14) and D22S929 (15,16) were chosen from the literature and professional gene databases (EntrezGene *http://www.ncbi.nlm.nih.gov/*). Heterozygous samples were visualized on Spreadex EL 400 gels (Elchrom Scientific, Cham, Switzerland), stained with SyberGold (Molecular Probes, Leiden, The Netherlands) and on 15% polyacrylamide gels, stained with silver. Absence or a significant decrease in the intensity of one of the D22S444 and D22S929 alleles in tumor, as compared with the autologous blood sample, was considered as LOH of *NF2* gene.

## Results

**Figure 1:**
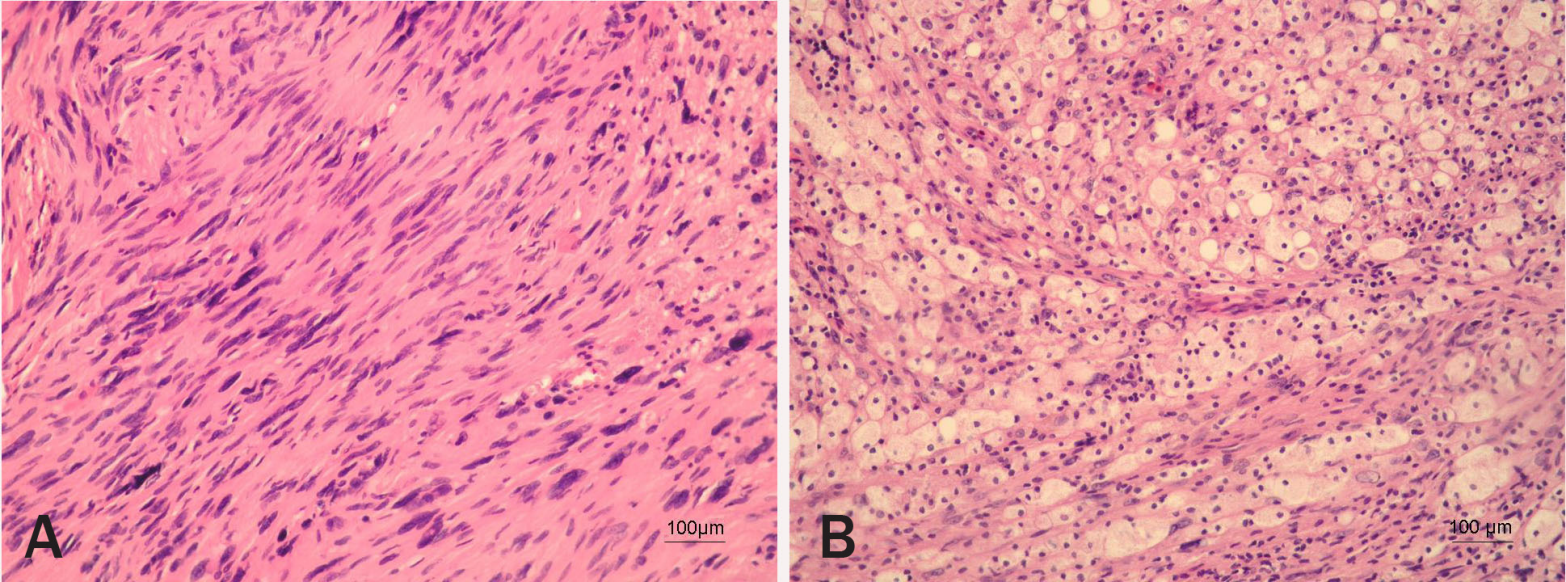
Vestibular schwannoma, (**A**) Antoni A. Tumors were composed of compact spindle cells that had twisted nuclei and indistinct cytoplasmic borders. They were arranged in short bundles. Nuclear palisading and Verocay bodies were present. (**B**) Antoni B. The tumor was composed of loosely arranged Schwann cells admixed with foamy macrophages. In some tumor cells degenerative nuclear changes were seen but mitotic activity was not observed (200 × , hematoxylin and eosin).

Schwannomas were classified as WHO grade I and specified as Antoni A or Antoni B (17) patterns (Figure 1 A and B). Seven (35%) were Antoni A, 9 (45%) were Antoni B, and 4 (20%) had mixed Antoni A and Antoni B features.

Our data set consisted of 20 patients, 15 female. The age of the patients ranged from 12 to 67 years (mean age 50.95; median 52.50). The mean age at diagnosis was 33 years for men and 57 years for women. Their symptoms lasted between 2 to 72 months (mean 38.95; median 42.0).

**Figure 2:**
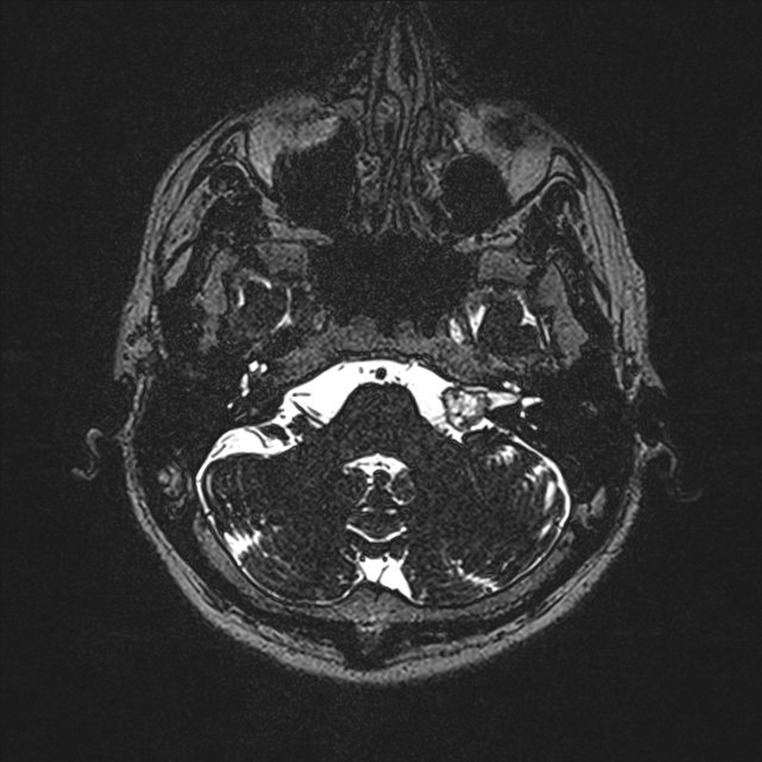
Magnetic resonance image showing lesion in the left pontocerebellar angle.

The localization of the tumor was as follows: 11 were left vestibular (50%) (Figure 2), 6 were right vestibular (25%), one was found in the right temporal region (IX nerve), while two were found in the left spinal LI and LII nerves. Intracranial schwannomas were predominantly found in women.

Of 20 schwannoma samples, 16 were informative when analyzed with both NF2 gene markers (80%). Eleven (55%) were informative for D22S444 and 14 (70%) for D22S929 microsatellite marker. The results regarding *NF2* gene showed 7 out of 16 heterozygous patients with allelic losses (43.75%). This is the total number of changes analyzed by both microsatellite markers. When specifying changes to distinct gene regions, there were 5 LOHs discovered with D22S444 (45.5%) and 2 LOHs discovered with D22S929 (14.3%). The LOHs were lost for one marker and not the other. D22S929 is an intragenic marker in intron 1 of the *NF2* gene, so our results showed that in patients heterozygous for D22S929 with loss of the distal marker D22S444 the deletion would start somewhere downstream of the first exons.

**Figure 3:**
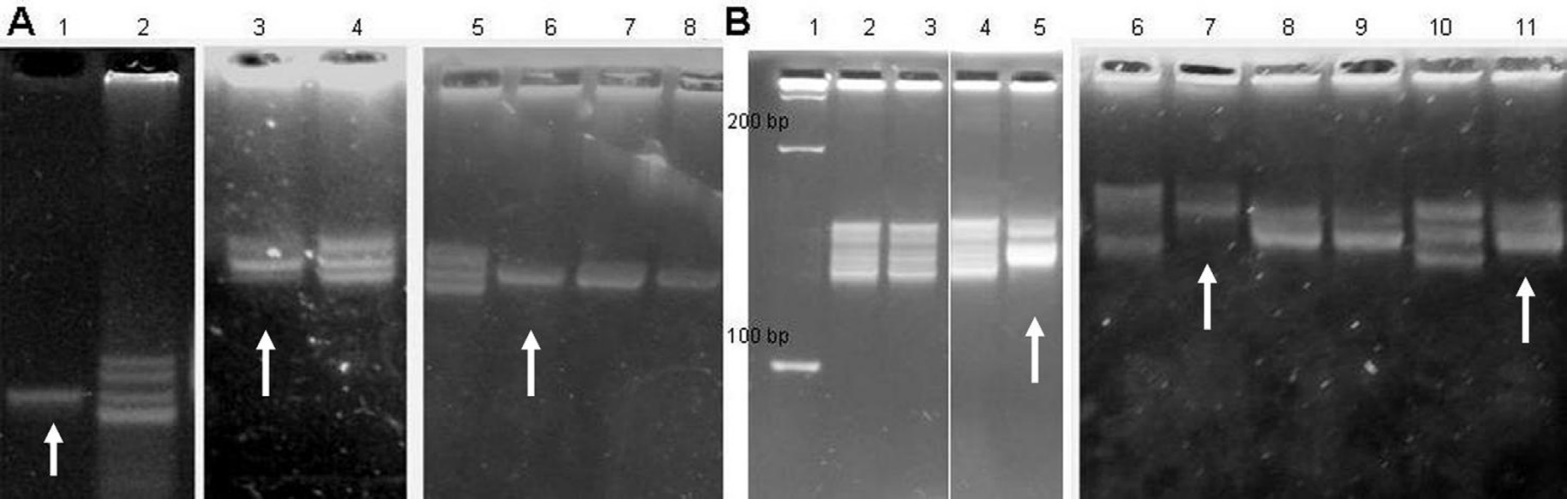
Losses of heterozigosity (LOHs) of the *NF2* gene in schwannomas. (**A**) Lanes 1, 3, 6 – samples demonstrating LOH at D22S444; lanes 2, 4, 5 –corresponding blood samples; lanes 7, 8 – uninformative patient. (**B**) Lane 1 –100 bp DNA standard; lanes 5, 7, 11 –samples demonstrating LOH at D22S929; lanes 4, 6, 10 – corresponding blood samples; lanes 2, 3 – heterozygous patient without LOH; lanes 8, 9 – uninformative patient.

LOHs of the *NF2* gene, revealed by both markers, are shown in Figure 3A (D22S444) and B (D22S929).

When assigning the gross deletions of the *NF2* gene to a specific pathohistological classification, the distribution of LOHs was as follows: 4 LOHs were found in Antoni B tumors (57%), 2 in Antoni A (29%), and 1 in Antoni A and B tumors (14%). The pathohistologic diagnosis of the analyzed samples, along with the LOH of the *NF2* gene and polymorphic status of both microsatellite markers is shown in Table 2.

**Table 2:** The analyzed samples, loss of heterozygosity of the *NF2* gene, and polymorphic status of D22S444 and D22S929 markers

Patient No.	Localization	Antoni	D22S444	D22S929	Symptoms/ months	Sex	Age
1	VIII* left	B	Heterozygous	Heterozygous	72	F	64
2	Spinal LII left	A	Homozygous	Homozygous	3	M	40
3	VIII right	B	LOH	Homozygous	42	F	50
4	VIII left	A	Homozygous	Homozygous	5	F	53
5	VIII right	B	Homozygous	Homozygous	36	F	62
6	VIII left	B	Heterozygous	LOH	24	F	33
7	VIII left	B	LOH	Heterozygous	54	F	51
8	VIII left	A	Homozygous	LOH	66	M	26
9	VIII right	A	LOH	Homozygous	42	M	12
10	VIII right	B	Homozygous	Heterozygous	36	F	63
11	IX right	A	Homozygous	Homozygous	36	F	59
12	VIII left	A + B	Homozygous	Heterozygous	30	F	52
13	VIII left	B	LOH	Heterozygous	60	F	67
14	VIII right	B	Homozygous	Heterozygous	54	F	51
15	VIII left	B	Heterozygous	Heterozygous	66	F	67
16	VIII left	A+B	Heterozygous	Heterozygous	48	F	67
17	Spinal LI left	A	Heterozygous	Heterozygous	2	F	49
18	VIII left	A+B	LOH	Heterozygous	48	M	60
19	VIII left	A	Heterozygous	Heterozygous	48	M	27
20	VIII right	A+B	Homozygous	Heterozygous	7	F	66

*VIII = eighth cranial nerve in the cerebellopontine angle (vestibular cranial schwannoma).

## Discussion

Our analysis using two microsatellite markers found 43.75% samples with gross deletions of the *NF2* gene. We have already mentioned that gross deletions of the *NF2* gene are frequent events in the molecular pathology of sporadic schwannoma (11). Although it is well known that the main cause for transformation of the Schwann cells into schwannomas is the inactivation of the *NF2* gene, and the consecutive loss of its protein merlin, the intracellular mechanism of this transformation still needs to be elucidated. It seems that the inactivation of the second allele often occurs via a large deletion of the 22q chromosomal region.

The investigated marker D22S929 is an intragenic marker located within the 32.2-kb-long intron 1 of the *NF2* gene (15,16). It is a dinucleotide repeat (15) with reported heterozygosity of 83%, while our sample showed the heterozygosity of 70%. The genetic alterations were found in the *NF2*'s intron, indicating intragenetic target of this deletion. The marker D22S444 is a tetranucleotide repeat proximal to the *NF2* gene, with reported informativity of 79% (18). The rates of allelic loss were different between the markers used. This variability of LOHs found in those two genetic regions could indicate more precisely the position of the deleted part and the size this deletion encompasses in our cases. The variability of the results obtained by different microsatellite markers can also elucidate the involvement of genomic instability regarding DNA replication and postreplication repair.

This type of analysis has never previously been performed on a cohort of patients from Croatia, and we believe that our results could broaden the overall *NF2* mutational spectrum. Our results are in accordance with the frequency of LOHs in sporadic schwannomas reported by other authors (19,20). In other studies, the frequency of LOHs in schwannomas was from 40%-80% – a fairly large range – which depended on the number of genetic markers used and the number of cases examined (19-26). An incidence of 42.6% of LOHs assessed by 4 microsatellite markers, a number that is very similar to our result, was reported in the study by Bian et al (19). Moreover, the same study reported difference in *NF2* LOHs between vestibular and spinal schwannomas and the association of higher proliferative index to the schwannomas showing LOH. Hadfield et al (20) reported LOH occurrence in 54 out of 96 (56%) sporadic vestibular schwannomas, a frequency that while clearly higher is not significant (χ^2^ = 0.433). There are reports of even higher frequencies of losses (22), which detected 72% of *NF2* deletions by direct sequencing and 77% of LOHs (23). Vestibular schwannomas were also analyzed by comparative genomic hybridization in several studies. Loss on 22q was reported in 23% of sporadic schwannomas (24), in which case it was slightly more common in tumors associated to NF2 than those found in sporadic cases, but this difference was not significant. Moreover, Warren et al (25) examined 66 sporadic vestibular schwannomas and found 23.7% of losses; while Koutsimpelas et al (26) found losses on the chromosome 22 in 30% of the cases. Mantripragada et al (21) performed a high resolution study using an array covering 1/3 of human chromosome 22 and found LOH in 45% of schwannomas. An important feature common to all of these analyses is the relatively small number of cases. As systematized in a meta-analysis of 12 years of studying of the mutational spectrum of *NF2* gene (8), it is obvious that the overall number of investigated sporadic cases remains rather small. Our sample therefore represents approximately 8% of the total worldwide sample size, and is the first that is derived from southeastern Europe.

Loss of expression of NF2 protein product merlin is a universal finding in all schwannomas examined, indicating inactivation of both NF2 alleles. The loss of immunoreactivity was reported in many studies (23,24,27). The main characteristic of cells lacking NF2 protein product is the loss of contact inhibition of proliferation (28). Associated with the loss of contact inhibition, merlin-lacking cells are also known to contain defective adherens junctions (29). Thus, it is understandable that cells that suffered merlin loss show deregulated adhesion to extracellular matrix, which is also shown in schwannoma cells. Furthermore, merlin seems to be directly involved in cytoskeletal organization relevant to myelination (30). Recent research on *NF2* gene has demonstrated that merlin is a tumor suppressor capable of modulating a wide range of signaling pathways that influence cell growth, motility, and apoptosis (30). It is clear that merlin is involved in different signal transduction pathways, Hippo and Ras/Raf/Mek pathways being the best characterized, while the latest reports also suggest merlin’s connection to the wnt signaling pathway (31,32). It has been shown that merlin’s inactivation is involved in about half of sporadic meningiomas, too. In our previous investigation on meningiomas, two LOHs of the *NF2 *gene were found with the D22S929 marker (33).

When assigning the gross deletions of the *NF2* gene to a specific pathohistological classification, in our study the distribution of LOHs was not associated to any particular morphology. As schwannomas are benign tumors that respond poorly to classical chemotherapeutics and often result in morbidity, the current therapies of choice are surgery and radiosurgery, but it is equally important to develop novel therapeutic approaches. In this context, understanding how merlin’s loss causes tumorigenesis would in all likelihood open the door for new therapies. As intracranial schwannomas are relatively rare, our sample represents a valuable asset to the analysis of *NF2* gross deletions in intracranial sporadic cases.

In conclusion, the frequency of NF2 allelic losses observed in Croatian patients is broadly similar to that reported in other populations and thus both confirms the existing hypothesis regarding the tumorigenesis of schwannomas, and contributes to schwannoma genetic profile, helping us to better understand its etiology and treatment.

## References

[1] Hanemann CO, Evans DG (2006). News on the genetics, epidemiology, medical care and translational research of Schwannomas.. J Neurol.

[2] Ghosh A, Talwar OP, Pradhan SV (2010). Tumour and tumour-like conditions of peripheral nerve origin: ten years' experience.. Kathmandu Univ Med J (KUMJ).

[3] Sughrue ME, Yeung AH, Rutkowski MJ, Cheung SW, Parsa AT (2011). Molecular biology of familial and sporadic vestibular schwannomas: implications for novel therapeutics.. J Neurosurg.

[4] Evans DG, Huson SM, Donnai D, Neary W, Blair V, Teare D (1992). A genetic study of type 2 neurofibromatosis in the United Kingdom. I. Prevalence, mutation rate, fitness, and confirmation of maternal transmission effect on severity.. J Med Genet.

[5] Antinheimo J, Sankila R, Carpen O, Pukkala E, Sainio M, Jaaskelainen J (2000). Population-based analysis of sporadic and type 2 neurofibromatosis-associated meningiomas and schwannomas.. Neurology.

[6] Gutmann DH, Geist RT, Xu H, Kim JS, Saporito-Irwin S (1998). Defects in neurofibromatosis 2 protein function can arise at multiple levels.. Hum Mol Genet.

[7] Fong B, Barkhoudarian G, Pezeshkian P, Parsa AT, Gopen Q, Yang I (2011). The molecular biology and novel treatments of vestibular schwannomas.. J Neurosurg.

[8] Ahronowitz I, Xin W, Kiely R, Sims K, MacCollin M, Nunes FP (2007). Mutational spectrum of NF2 gene: A meta-analysis of 12 years of research and diagnostic laboratory findings.. Hum Mutat.

[9] Celis-Aguilar E, Lassaletta L, Torres-Martin M, Rodrigues FY, Nistal M, Castresana JS (2012). The molecular biology of vestibular schwannomas and its association with hearing loss: a review. Genetics Research International.

[10] Irving RM, Harada T, Moffat DA, Hardy DG, Whittaker JL, Xuereb JH (1997). Somatic neurofibromatosis type 2 gene mutations and growth characteristics in vestibular schwannoma.. Am J Otol.

[11] Jacoby LB, MacCollin M, Barone R, Ramesh V, Gusella JF (1996). Frequency and distribution of NF2 mutations in schwannomas.. Genes Chromosomes Cancer.

[12] Hanemann CO (2008). Magic but treatable? Tumours due to loss of Merlin.. Brain.

[13] Den Bakker MA, van Tilborg AAG, Kros JM, Zwarthoff E (2001). Truncated NF2 proteins are not detected in meningiomas and schwannomas.. Neuropathology..

[14] Kros J, de Greve K, van Tilborg A, Hop W, Pieterman H, Avezaat C (2001). Lekane dit Deprez R, Zwarthoff E. NF2 status of meningiomas is associated with tumour localization and histology.. J Pathol.

[15] Bourn D, Strachan T (1995). Highly polymorphic dinucleotide repeat at the NF2 gene.. Hum Genet.

[16] Ueki K, Wen-Bin C, Narita Y, Asai A, Kirino T (1999). Tight association of loss of merlin expression with loss of heterozygosity at chromosome 22q in sporadic meningiomas.. Cancer Res.

[17] Scheithauer BW, Louis DN, Hunter S, Woodruff JM, Antonescu CR. Tumours of the cranial and Paraspinal Nerves. Schwannoma. In: Louis DN, Ohgaki H, Wiestler OD, Cavenee WK, editors. WHO classification of tumours of the central nervous system 4th edition. Lyon (France): International Agency for Research on Cancer; 2007. p.152-5.

[18] Lee JY, Finkelstein S, Hamilton RL, Rekha R, King JT, Omalu B (2004). Loss of heterozygosity analysis of benign, atypical, and anaplastic meningiomas.. Neurosurgery.

[19] Bian LG, Sun QF, Tirakotai W, Zhao WG, Shen JK, Luo QZ (2005). Loss of heterozygosity on chromosome 22 in sporadic schwannoma and its relation to the proliferation of tumor cells.. Chin Med J (Engl).

[20] Hadfield KD, Smith MJ, Urquhart JE, Wallace AJ, Bowers NL, King AT (2010). Rates of loss of heterozygosity and mitotic recombination in NF2 schwannomas, sporadic vestibular schwannomas and schwannomatosis schwannomas.. Oncogene.

[21] Mantripragada KK, Buckley PG, Benetkiewicz M, De Bustos C, Hirvelä C, Jarbo C (2003). High-resolution profiling of an 11 Mb segment of human chromosome 22 in sporadic schwannoma using array-CGH.. Int J Oncol.

[22] Aarhus M, Bruland O, Sćtran HA, Mork SJ, Lund-Johansen M, Knappskog PM (2010). Global gene expression profiling and tissue microarray reveal novel candidate genes and down-regulation of the tumor suppressor gene CAV1 in sporadic vestibular schwannomas.. Neurosurgery.

[23] Lee DJ, Maseyesva B, Westra W, Long D, Niparko JK, Califano J (2006). Microsatellite analysis of recurrent vestibular Schwannoma (acoustic neuroma) following stereotactic radiosurgery.. Otol Neurotol.

[24] Antinheimo J, Sallinen SL, Sallinen P, Haapasalo H, Helin H, Horelli-Kuitunen N (2000). Genetic aberrations in sporadic and neurofibromatosis 2 (NF2)-associated Schwannomas studied by comparative genomic hybridization (CGH).. Acta Neurochir (Wien).

[25] Warren C, James LA, Ramsden RT, Wallace A, Baser ME, Varley JM (2003). Identification of recurrent regions of chromosome loss and gain in vestibular schwannomas using comparative genomic hybridization.. J Med Genet.

[26] Koutsimpelas D, Felmeden U, Mann WJ, Brieger J (2011). Analysis of cytogenetic aberrations in sporadic vestibular schwannoma by comparative genomic hybridization.. J Neurooncol.

[27] Hitotsumatsu T, Iwaki T, Kitamoto T, Mizoguchi M, Suzuki SO, Hamada Y (1997). Expression of neurofibromatosis 2 protein in human brain tumors: an immunohistochemical study.. Acta Neuropathol.

[28] Okada T, Lopez-Lago M, Giancotti FG (2005). Merlin/NF-2 mediates contact inhibition of growth by suppressing recruitment of Rac to the plasma membrane.. J Cell Biol.

[29] Lallemand D, Curto M, Saotome I, Giovannini M, McClatchey AI (2003). NF2 deficiency promotes tumorigenesis and metastasis by destabilizing adherens junctions.. Genes Dev.

[30] Stamenkovic I, Yu Q (2010). Merlin, a “magic” linker between the extracellular cues and intracellular signaling pathways that regulate cell motility, proliferation, and survival.. Curr Protein Pept Sci.

[31] Morrison H, Sperka T, Manent J, Giovannini M, Ponta H, Herrlich P (2007). Merlin/neurofibromatosis type 2 suppresses growth by inhibiting the activation of Ras and Rac.. Cancer Res.

[32] Bosco EE, Nakai Y, Hennigan RF, Ratner N, Zheng Y (2010). NF2-deficient cells depend on the Rac1-canonical Wnt signaling pathway to promote the loss of contact inhibition of proliferation.. Oncogene.

[33] Pecina-Slaus N, Nikuseva Martic T, Deak AJ, Zeljko M, Hrascan R, Tomas D (2010). Genetic and protein changes of E-cadherin in meningiomas.. J Cancer Res Clin Oncol.

